# The short-term outcome of intracranial stenosis with distal thrombosis treated with balloon-assisted tracking

**DOI:** 10.3389/fneur.2024.1308152

**Published:** 2024-02-16

**Authors:** Hui Li, Xiangyu Meng, Kai Mao, Linlin Liu, Lifeng Xu, Lingyou Chen, Can Xu, Wenqing Wang, Conghui Li

**Affiliations:** The First Hospital of Hebei Medical University, Shijiazhuang, China

**Keywords:** stroke, intracranial atherosclerotic disease (ICAD), balloon-assisted tracking (BAT) technique, mechanical thrombectomy (MT), intracranial stenosis

## Abstract

**Background and purposes:**

Treating intracranial stenosis with distal thrombosis (IS&DT) using traditional mechanical thrombectomy (MT) techniques has proven challenging. This study aimed to summarize the experience of utilizing the balloon-assisted tracking (BAT) technique for IS&DT.

**Methods:**

Demographic and morphologic characteristics of patients with IS&DT were collected for this study. The BAT technique, involving a half-deflated balloon outside the intermediate catheter tip, was used in all patients to navigate through the proximal stenosis. Various parameters were recorded, including the sequence of vascular reperfusion, the puncture-to-reperfusion time (PRT), the residual stenosis rate, and the occurrence of re-occlusion. The thrombolysis in cerebral infarction (TICI) scale was used to assess the reperfusion of intracranial vessels, with a TICI score of ≥2b considered as successful perfusion. The clinical status of patients was evaluated at three time points: pre-procedure, post-procedure, and at discharge using the modified Rankin score (mRS).

**Results:**

In this study, a total of 10 patients were diagnosed with IS&DT, consisting of 9 male patients (90.0%) and 1 female patient (10.0%). The patients’ mean age was 63.10 years (ranging from 29 to 79 years). The mean National Institute of Health Stroke Scale (NIHSS) score before treatment was 24.3 (ranging from 12 to 40), indicating the severity of their condition. Following the procedure, all patients achieved successful reperfusion with a thrombolysis in cerebral infarction (TICI) score of ≥2b. The average puncture-to-reperfusion time (PRT) was 51.8 min (ranging from 25 to 100 min), indicating the time taken for the procedure. During the perioperative period, three patients (30.0%) experienced complications. One patient had hemorrhage, while two patients had contrast extravasation. Among these cases, only the patient with hemorrhage (10%) suffered from a permanent neurological function deficit. At discharge, the patient’s condition showed improvement. The mean NIHSS score decreased to 13.2 (ranging from 1 to 34), indicating a positive response to treatment. The mean mRS score at discharge was 3.2 (ranging from 1 to 5), showing some level of functional improvement.

**Conclusion:**

In conclusion, the use of the balloon-assisted tracking (BAT) technique for treating intracranial stenosis with distal thrombosis (IS&DT) showed promising results. However, a moderate rate of perioperative complications was observed, warranting further investigation and refinement of the procedure.

## Introduction

Intracranial stenosis with distal thrombosis (IS&DT) presents a challenging scenario for mechanical thrombectomy (MT) procedures due to the simultaneous occurrence of proximal stenosis related to intracranial atherosclerosis (ICAS) and distal thrombosis. This condition can result in delayed or failed access construction and lead to poor prognoses, particularly when ICAS-related stenosis occurs in intracranial vessel (1–5). Addressing this tandem lesion requires different techniques for acute thrombosis, stroke, and intracranial stenosis, but the lack of established studies and paradigms has hindered effective solutions. However, previous studies have shown promising results using the balloon-assisted tracking (BAT) technique for treating extracranial ICA stenosis and acute distal vessel occlusion, rapidly and safely restoring cerebral blood flow while addressing proximal stenosis simultaneously, thus alleviating economic and physiological burdens on patients requiring reoperation (6).

The BAT technique, initially introduced by interventional cardiologists, involves using a partially deflated balloon exposed at the catheter tip to facilitate passage through stenosed arterial segments. Recent case studies have suggested the feasibility and potential applicability of this technique in the field of neurointerventions as well (7–10). Indeed, the application of the BAT technique to IS&DT cases has been reported as rare. Therefore, the purpose of this study was to summarize the experience of using the BAT technique specifically for treating IS&DT. By providing comprehensive details and insights, the study aimed to promote the wider adoption and utilization of this technique in such challenging situations.

## Methods

### Patients

Patients with a tandem lesion consisting of intracranial ICAS-related stenosis and distal thrombosis were enrolled in our institution. Informal consent was obtained from each patient or their relatives for permission. The institutional review board approved the study. IS&DT is characterized by an acute occlusion of the distal cerebral artery, concomitant with atherosclerosis-related stenosis in the proximal intracranial segment. The inclusion criteria for this study are as follows: 1. patients aged >18 years and < 80 years; 2. patients experiencing acute ischemic stroke onset within 6 h, with NIHSS >6, ASPECT >6 for anterior circulation occlusion and pc-ASPECT >6 for posterior circulation occlusion, or eligible for DAWN or DEFUSE III treatment based on imaging assessment; for vertebral-basilar artery occlusion, treatment could be performed within 24 h; 3. the presence of intracranial ICAS confirmed by clinical presentation, preoperative CTA, and intra-operative microcatheter first-pass effects or prior evidence of associated stenosis; and 4. all patients underwent the BAT technique during the procedure. The materials used for establishing vascular access were recorded, and various morphological features were assessed, including the vascular system involved, the sequence of vascular reperfusion, puncture-to-reperfusion time, residual stenosis rate, and re-occlusion occurrence. The thrombolysis in cerebral infarction (TICI) scale was used to evaluate intracranial vessel reperfusion, with a TICI score ≥ 2b indicating successful perfusion.

### Data collection

Upon admission, a head CT scan was conducted, both at the time of admission and immediately after the procedure. Additionally, a CT angiography image was obtained to confirm the structure of the cerebral vascular system before performing a thrombectomy. Demographic data, such as age, sex, onset symptoms, and relevant medical history, including hypertension, atrial fibrillation, diabetes, and cigarette smoking history, were recorded. Clinical parameters, such as the National Institute of Health Stroke Scale (NIHSS) score, thrombolysis in cerebral infarction (TICI) scale, time from symptom onset to hospital arrival, time from puncture to reperfusion, and the location of occlusion at admission, were also documented. The study also registered details of the mechanical thrombectomy procedure, including the sequence of vascular reperfusion, the materials used to establish vascular access, and the rate of residual stenosis. Digital subtraction angiography (DSA) performed before and after the treatment was reviewed and evaluated by two experienced neurosurgeons to determine the TICI grades before and after recanalization.

The evaluation indices for assessing the success of the procedure and short-term outcomes were as follows:

1. Rate of definitive intracranial revascularization: this indicates the success rate of restoring blood flow in the occluded intracranial vessels.
2. TICI grade before and after treatment: the thrombolysis in cerebral infarction (TICI) scale was used to assess the degree of reperfusion in the intracranial vessels before and after the treatment.
3. Procedural-induced complications: this records any complications or adverse events that occurred during the procedure itself.
4. Postprocedural adverse events: this includes any complications or adverse events that occurred after the procedure, such as parenchymal reperfusion hematoma or contrast extravasation.

Clinical status was assessed at three different time points:

1. Pre-procedure: this involves evaluating the patient’s condition before the thrombectomy procedure using the National Institutes of Health Stroke Scale (NIHSS) score, Glasgow Coma Scale (GCS), and modified Rankin Scale (mRS).
2. Post-procedure: the patient’s condition was reassessed immediately after the thrombectomy procedure using the same clinical scales (NIHSS, GCS, and mRS).
3. Discharge: the patient’s clinical status was evaluated again at the time of discharge using the NIHSS score, GCS, and mRS to assess the short-term outcome after the procedure.

### Endovascular treatment

Each patient undergoing endovascular treatment was administered general anesthesia. Sterilization of both groins was carried out for interventional therapy. An 8F sheath was introduced into the femoral artery, and 5,000 IU of unfractionated heparin was administered. No antiplatelet therapy was administered prior to treatment in these cases. After the treatment and a follow-up CT scan showed no intracranial hemorrhage, the patients received an intravenous continuous infusion of tirofiban for 24 h. Twelve hours after the infusion, aspirin 100 mg and clopidogrel 75 mg were prescribed orally until the next month’s follow-up. For patients who underwent stent placement, they were required to continue oral medication for 3 months before any medication adjustment. Diagnostic angiography was conducted using an angiographic catheter. Following angiography, the occluded vessel suspected to be caused by intracranial atherosclerosis (ICAS) was confirmed. Subsequently, the angiographic catheter was replaced with an 8F guiding catheter, which was guided to approach the site of intracranial ICAS occlusion. This approach included navigating to the vertebral artery (VA) V4 segment, basilar artery (BA), internal carotid artery (ICA) C4 segment or above, and middle cerebral artery (MCA). A coaxial microcatheter guided by a microwire was advanced to the proximal occlusion site. Careful advancement was conducted to establish patency in the distal artery, followed by the retrieval of the microcatheter. If the microwire encountered resistance during this process, an undersized balloon could be employed for pre-expansion, facilitating the passage of the microwire and microcatheter. Guided by the guiding catheter, angiography was performed to assess the flow through the occlusion site. The “first-pass effect” of the microcatheter was verified, confirming the ICAS-related occlusion. The balloon-assisted tracking (BAT) technique was employed in cases where a distal thrombus was obstructing vessel flow concurrently.

**Figure 1:**
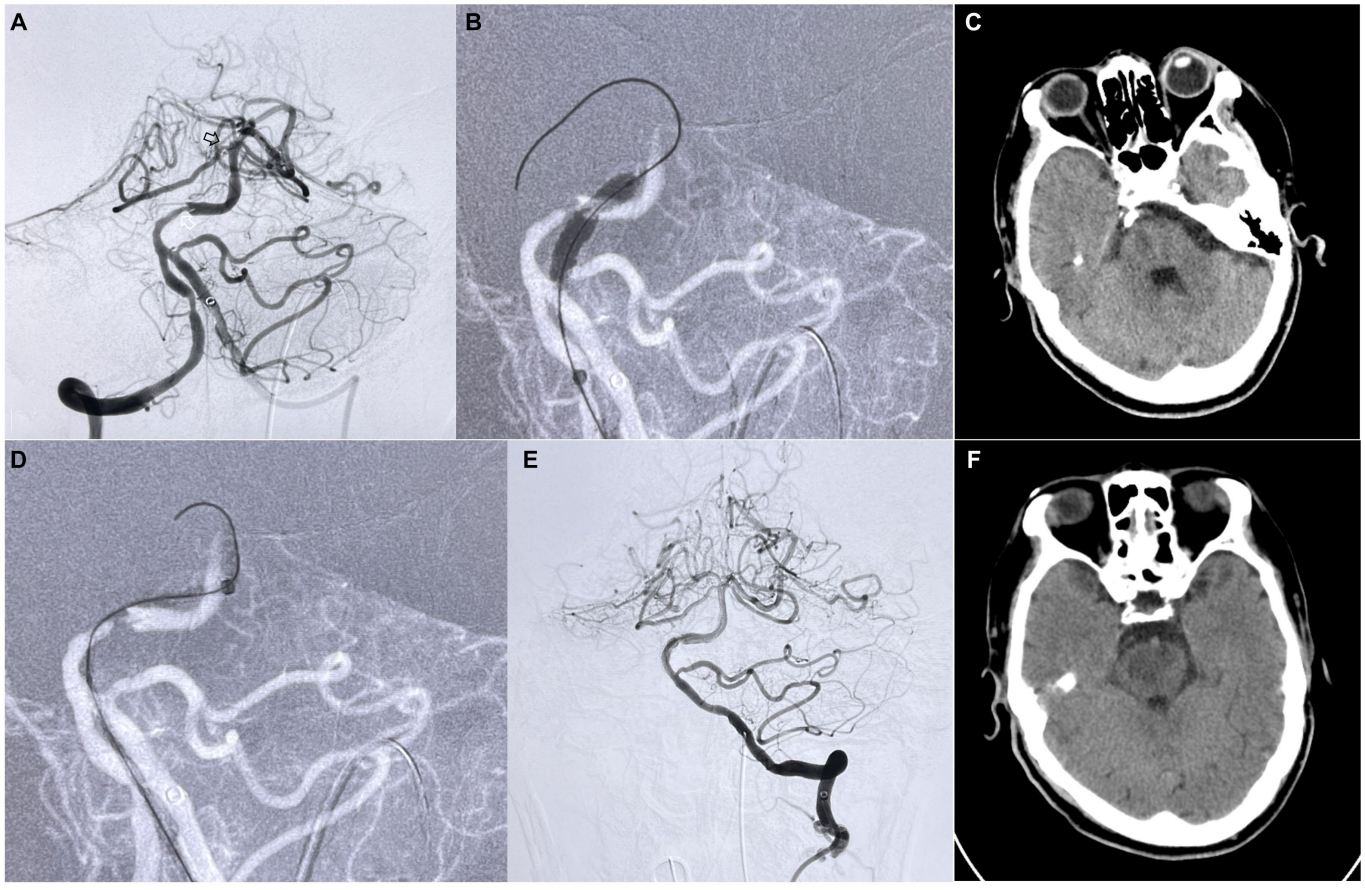
Patient suffered a sudden weakness of both lower limbs and loss of consciousness for a last-known normal time of 17 h and was admitted to the hospital. **(A)** Angiography shows an ICAS-related stenosis located at the proximal of the basilar artery (white arrow) accompanied by a distal thrombosis occluded at the top of the basilar artery (black arrow). **(B,D)** The standard BAT technique was applied to this patient for crossing the narrow site proximal to the intermediate catheter. **(E)** After thrombectomy for the distal acute occlusion using a Solitaire stent, the proximal stenosis was only treated with balloon dilation to achieve a TICI 2b recanalization. **(C,F)** Due to the long-term occlusion, compared with preoperative CT **(C)**, postoperative CT **(F)** shows that the infarction area focus of the midbrain and pons was larger than before. No signs of intracranial hemorrhage or massive infarction were found after BAT and thrombectomy.

The recanalization of the proximal ICAS-related occlusion was performed using a slightly shaped 0.014 microwire protruding outside an undersized balloon. The wire was carefully advanced across the proximal ICAS-related occlusion. Careful attention was taken to keep the wire in a true lumen by positioning it through the tiny gap of the ICAS-related stenosis identified on initial angiography. The undersized balloon was navigated over the microwire, immediately covering the ICAS lesion. The balloon was then partially inflated to approximately 60% of its maximal volume. After a while, the balloon was partially deflated, and the guide catheter was advanced to partially cover it. A streamlined outline of the balloon-catheter tip complex was formed with the half-deflated balloon outside from the guiding catheter tip, and the razor effect created by the gap at the tip of the catheter was also eliminated. The system was then gently advanced together through the plaque to prevent the rupture and dislodgment of the plaque. After positioning the guiding catheter, the blood flow through the plaque was blocked temporarily, and the balloon was deflated and removed. A microcatheter was exchanged to perform mechanical thrombectomy using a combined technique including stent retrieval with local aspiration for complete reperfusion for the distal occlusion. Figure 1 shows a patient with a basilar artery tandem lesion using the BAT technique to overcome the stenosis and applying thrombectomy thereafter at the distal vessel.

### Statistical analysis

The Pearson *χ*^2^ or Fisher exact analysis was used to compare categorical variables. The Student’s t-test or Mann–Whitney U-test was used to compare continuous variables based on different outcomes. The data for categorical variables are described in absolute terms.

Frequencies and the data for continuous variables were given as the mean and standard deviation. All statistical analyzes were performed with IBM SPSS Statistics 20.0.

## Results

Ten patients diagnosed with IS&DT underwent treatment utilizing the BAT technique to recanalize occluded vessels. The study group consisted primarily of male patients (9 males) with a mean age of 63.10 years, ranging from 29 to 79 years. The average duration from symptom onset to hospital admission was 5.8 h, ranging from 3 to 17 h. Patients undergoing mechanical thrombectomy (MT) beyond the standard time window (6–16 h and 16–24 h) underwent preoperative CT perfusion (CTP) and CT angiography (CTA) assessments. The core infarct area and the ischemic penumbra region in these patients met the inclusion criteria outlined in the DAWN and DEFUSE-3 studies. This study also documented the initial onset symptoms of the patients. Among the participants, nine patients exhibited weakness in the lateral limbs, five had dysarthria, four showed progressive deterioration of consciousness, one presented with visual field impairment, and two experienced dysphagia. Additionally, seven patients had a history of hypertension, four had previously suffered from cerebral infarction, and one had a history of atrial fibrillation.

Among these 10 patients, 3 experienced vascular occlusions within the anterior circulation system of the internal carotid artery, while the remaining 7 faced acute vascular occlusions within the intracranial segment of the vertebral-basilar system artery. Upon admission, the patients exhibited an average NIHSS score of 24.3 (ranging from 12 to 40) and a mean mRS score of 4 (ranging from 2 to 5). In an effort to expedite recanalization of the intracranial artery, all patients underwent the balloon-assisted tracking (BAT) technique, with a mean puncture-to-reperfusion time (PRT) of 51.8 min (ranging from 25 to 100 min), following a comprehensive evaluation by two experienced neurosurgeons. The clinical characteristics relevant to each patient, along with the specific lesion locations, are outlined in Table 1.

**Table 1:** Characteristics of enrolled patients.

Characters	Values
Total patients	10
Gender (*n*, %)	
Male	9 (90)
Female	1 (10)
Age (years)	63.10 (29–79)
Initial presentations (*n*, %)	
Weakness in the lateral limbs	9 (90)
Dysarthria	5 (50)
Worsening of consciousness	4 (40)
Visual field impairment	1 (10)
Dysphagia	2 (20)
Involved artery (*n*, %)	
System of internal carotid artery	3 (30)
System of the vertebral-basilar artery	7 (70)
NIHSS on admission (mean, range)	24.3 (12–40)
mRS on admission (mean, range)	4 (2–5)
Mean period from onset to hospital (hours, range)	5.8 (3–17)
Mean PRT^*^ (min, range)	51.8 (25–100)
NIHSS at discharge (mean, range)	13.2 (1–34)
mRS at discharge (mean, range)	3 (1–5)
The mean length of hospitalization (mean, days)	15.3
The mean increase in NIHSS	11.1
The mean decrease in mRS	1.1

PRT, puncture-to-reperfusion time.

Among the entire cohort of enrolled patients, no instances of failed mechanical thrombectomy (MT) procedures or unsuccessful proximal stenosis treatments were observed. In every patient, immediate post-treatment cerebral angiography revealed substantial restoration of blood flow within the target vessel, indicating a significant improvement in thrombolysis in cerebral infarction (TICI) scores.

During the perioperative phase, complications arose in three patients (30%). One of these patients experienced postoperative hemorrhagic transformation, leading to a notable decline in neurological function and an increase in the postoperative modified Rankin Scale (mRS) score from 2 to 5. At the time of discharge, only this patient (10%) who had suffered postoperative hemorrhage demonstrated a permanent neurological deficit. The remaining two patients exhibited contrast medium extravasation following treatment, which was identified through postoperative CT scans. Swift clinical and CT-guided interventions, including anti-epilepsy measures, dehydration, and rehydration, facilitated a rapid recovery from clinical symptoms, with evident improvement in CT scans. Symptomatic relief was progressively achieved in these patients through conservative management.

Among all these patients, the mean length of hospitalization was 15.3 days. After treatment, the mean NIHSS score of patients at discharge was 13.2 (1–34), with a mean decrease of 11.1 in NIHSS score at discharge compared with preoperative; only one patient had an increase in NIHSS score at discharge, who suffered the postprocedure hemorrhage mentioned before; the mean mRS score at discharge was 3.2 (1–5), with a mean decrease of 1.1 in mRS at discharge compared with preoperative. Only the above-mentioned patient with bleeding had an increase in the mRS score after interventional treatment.

## Discussion

Patients with IS&DT, characterized by both proximal intracranial atherosclerosis-related stenosis and acute occlusion of distal vessels, present a complex and challenging clinical scenario. The treatment of this situation has seen significant advancements with the evolution of materials and interventional techniques. These innovations and insights have greatly improved the management and outcomes of such cases. The challenges associated with conventional mechanical thrombectomy in patients with IS&DT, particularly navigating the microcatheter through the stenosis and successfully deploying stents, are well-recognized difficulties. This complexity is exacerbated when both the proximal intracranial atherosclerosis-related stenosis and the distal stroke occur within the intracranial vessels, posing increased risks throughout the procedure. Addressing intracranial vessel stenosis necessitates the development of distinct access strategies and specialized techniques. The balloon-assisted tracking (BAT) technique, initially introduced in cardiovascular interventions, has found valuable application in cases where vasospasm complicates access during coronary angiography or treatment (11). This approach has inspired neurosurgeons and neurointerventionalists to apply it to the treatment of intracranial stenosis.

The balloon-assisted tracking (BAT) technique is characterized by partially releasing the balloon at the catheter’s tip, allowing a portion of the balloon to be exposed outside the catheter, and then inflating the balloon to, among other things, dilate the stenosis and eliminate the step-off effect between the access and the catheter. This innovative method enhances the efficiency and effectiveness of vessel recanalization and blood flow restoration. By utilizing the BAT technique, neurointerventionalists can achieve more rapid and efficient treatment strategies for patients with intracranial atherosclerosis (ICAS) combined with distal acute stroke. Compared with traditional treatment strategies, BAT is more suitable for this condition, allowing the intermediate catheter to pass through the stenosis more quickly and safely, reducing the risk of thrombus escape because the forward blood flow is blocked, and enabling the blood flow to recover more quickly. In this study, it is noteworthy that all patients had IS&DT lesions, which are particularly challenging cases. Interestingly, the mean puncture-to-reperfusion time (PRT) after employing the Balloon-assisted tracking (BAT) technique was recorded as 51.8 min. Importantly, this PRT was not significantly longer than what has been reported in previous literature for conventional techniques (12, 13). After undergoing endovascular treatment using the BAT technique, all patients experienced successful vessel recanalization, leading to significantly improved reperfusion compared to their preoperative condition. When applying the BAT technique to address intracranial atherosclerotic stenosis (ICAS) associated with distal acute stroke, the intermediate catheter effectively navigated through proximal stenoses, preventing the razor effect on the catheter’s rim and reducing the risk of fresh thrombus detachment, thereby blocking distal vessels. When the guiding catheter was navigated to the ICAS segment using the BAT technique, due to the close contact between the catheter and the plate at the stenosis site, the forward blood flow was blocked, so the balloon-guiding catheter was not used in this study (14). Notably, there were no complications directly attributable to the BAT technique. After the treatment of distal occlusion, stent implementation was performed in 7 out of 10 patients (70%) at proximal stenosis sites. Only one patient experienced a post-treatment consciousness deficit, but this patient initially presented with a high preoperative NIHSS score of 33 and had a tandem lesion in the vertebrobasilar system. Postoperative CT scans revealed no hemorrhage or extensive infarction, and postoperative DSA confirmed vascular recanalization. The patient’s postoperative CT indicated a focal infarction in the midbrain and pons. The decline in consciousness was attributed to the extended time from symptom onset to admission (17 h), as the core infarct area encompassed the brainstem. Postoperative edema in the infarct area worsened the situation (see Figure 1). Nevertheless, the patient’s NIHSS score improved at discharge compared to the preoperative assessment. In conclusion, the BAT technique proves to be effective and expeditious in recanalizing intracranial arteries affected by acute stroke-related ICAS stenoses. Additionally, treating proximal stenoses simultaneously does not significantly impact operative time or patients’ prognosis.

Jan-Karl et al. (7) reported a patient with intracranial stenosis using BAT to overcome the site of occlusion during thrombectomy for stroke. This is the first report of the adaptation of this technique to obtain distal access for thrombectomy in stroke. The author proposed that the application of BAT should be kept in mind during MT procedures when proximal ICAS stenosis impedes access to the distal occlusion site. In our study, we expanded the cohort to include patients with IS&DT, demonstrating the safety and efficacy of the BAT technique in this lesion. Our findings also underscore the efficiency of addressing proximal stenosis during a single session, ensuring optimal vascular recanalization without increasing procedural time. In a recent study involving 107 patients with tandem internal carotid occlusions, Csaba Nagy et al. (6) reported that successful recanalization of the ICA using the BAT technique was achieved in 100 (93%) and successful intracranial revascularization in 88 (82%) patients. There were no complications attributable to the BAT technique. Additional data from this study highlight the enhanced safety and efficacy of the BAT technique when used to treat tandem lesions. Though in this study, the proximal stenosis only involved the extracranial segment of ICA, it underscores the importance of considering BAT as a viable option for patients with tandem lesions, irrespective of whether the stenosis is intracranial or extracranial. The application of the BAT technique in IS&DT demonstrated feasibility and efficiency.

This single-center retrospective research has some limitations. First of all, the relatively small number of patients showed not enough to recognize complications during the BAT procedure; a larger cohort and a randomized design of research are needed to overcome this bias. Furthermore, this study presented a short-term outcome of tandem lesions treated with the balloon-assisted tracking (BAT) technique, and a longer period of follow-up is needed to confirm the long-term safety and efficacy of BAT technology in the treatment of these patients.

## Conclusion

The BAT technique is feasible for treating acute tandem thrombosis strokes associated with ICAS-related large vessel occlusion. The short-term safety and efficacy of this method have been preliminarily confirmed in our study.

## Data availability statement

The raw data supporting the conclusions of this article will be made available by the authors, without undue reservation.

## Ethics statement

The studies involving humans were approved by medical ethics committee of the first hospital of Hebei Medical University. The studies were conducted in accordance with the local legislation and institutional requirements. The participants provided their written informed consent to participate in this study.

## Author contributions

HL: Methodology, Supervision, Writing – review & editing, Conceptualization, Funding acquisition, Project administration. XM: Methodology, Supervision, Writing – review & editing, Formal analysis, Software, Writing – original draft. KM: Software, Writing – original draft. LL: Data curation, Investigation, Writing – review & editing. LX: Formal analysis, Writing – review & editing. LC: Methodology, Writing – review & editing. CX: Formal analysis, Writing – review & editing. WW: Supervision, Writing – original draft. CL: Conceptualization, Supervision, Writing – review & editing.
